# Supplementary material for: The Antioxidant Potential and Anticancer Activity of Halodule uninervis Ethanolic Extract against Triple-Negative Breast Cancer Cells
Source: Antioxidants (Basel). 2024 Jun 14;13(6):726. doi: 10.3390/antiox13060726 (PMC11200955; doi:10.3390/antiox13060726)
Supplement: Supplementary file 1 [file antioxidants-13-00726-s001.zip › antioxidants-2989109-supplementary.pdf]

Supplementary Tables and Figures

Table S1. The half-maximum inhibitory concentration (IC50) of *Halodule uninervis* ethanolic extract against different cancer cell lines at 24, 48, and 72 h.

	MDA-MB-231	Capan-2	HCT116	22RV1
24h	525.34 µg/ml	424.83 µg/ml	672.96 µg/ml	827.29 µg/ml
48h	173.37 µg/ml	277.24 µg/ml	245.21 µg/ml	153.72 µg/ml
72h	146.74 µg/ml	227.11 µg/ml	221.83 µg/ml	109.90 µg/ml

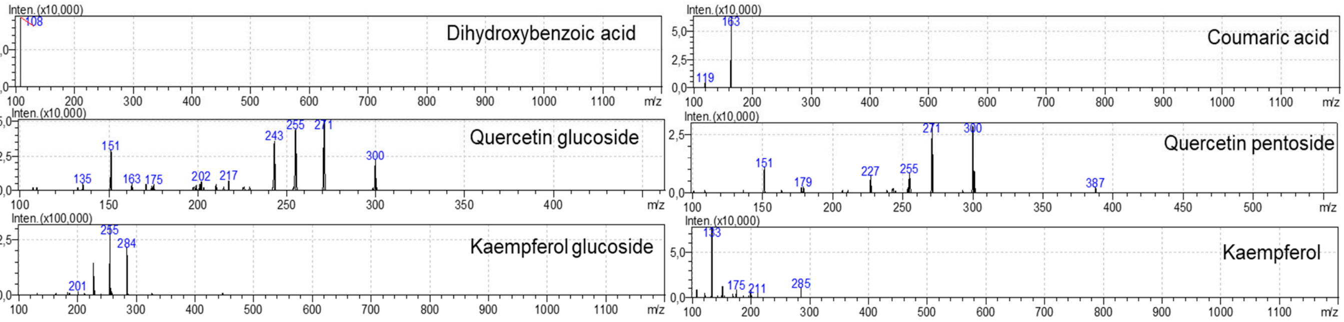

Figure S1. MS2 of some identified compounds in *Halodule uninervis* ethanolic extract.

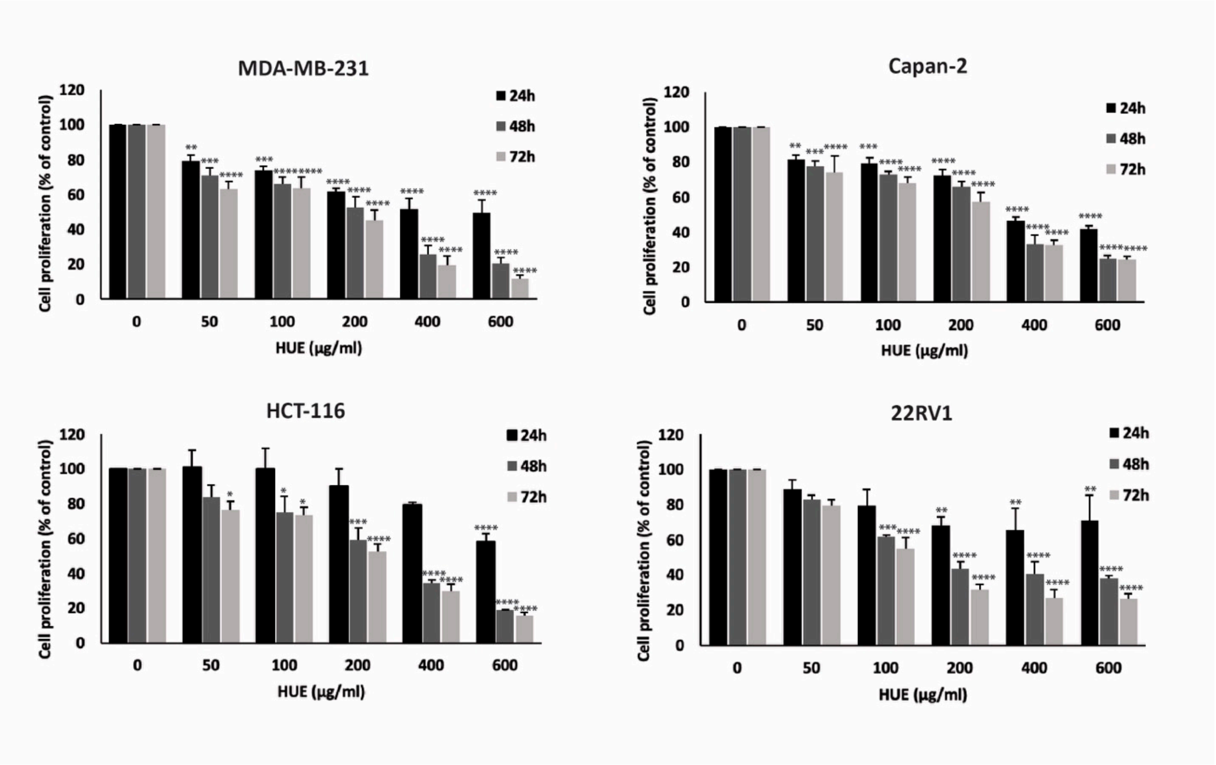

Figure S2. *Halodule uninervis* ethanolic extract exhibits an anticancer effect by inhibiting cellular proliferation of MDA-MB-231, Capan-2, HCT-116, and 22RV1 cell lines. Cells were treated with and without the indicated concentrations of

HUE for 24, 48, and 72 hours. 80% ethanol was used as the vehicle control. Viability was examined using the metabolic-dye-based MTT assay. Data represent the mean ± SEM of three independent experiments (n = 3) and are expressed as a percentage of the corresponding control cells. (*$p < 0.05$, **$p < 0.005$, *** $p < 0.001$, **** $p < 0.0001$).

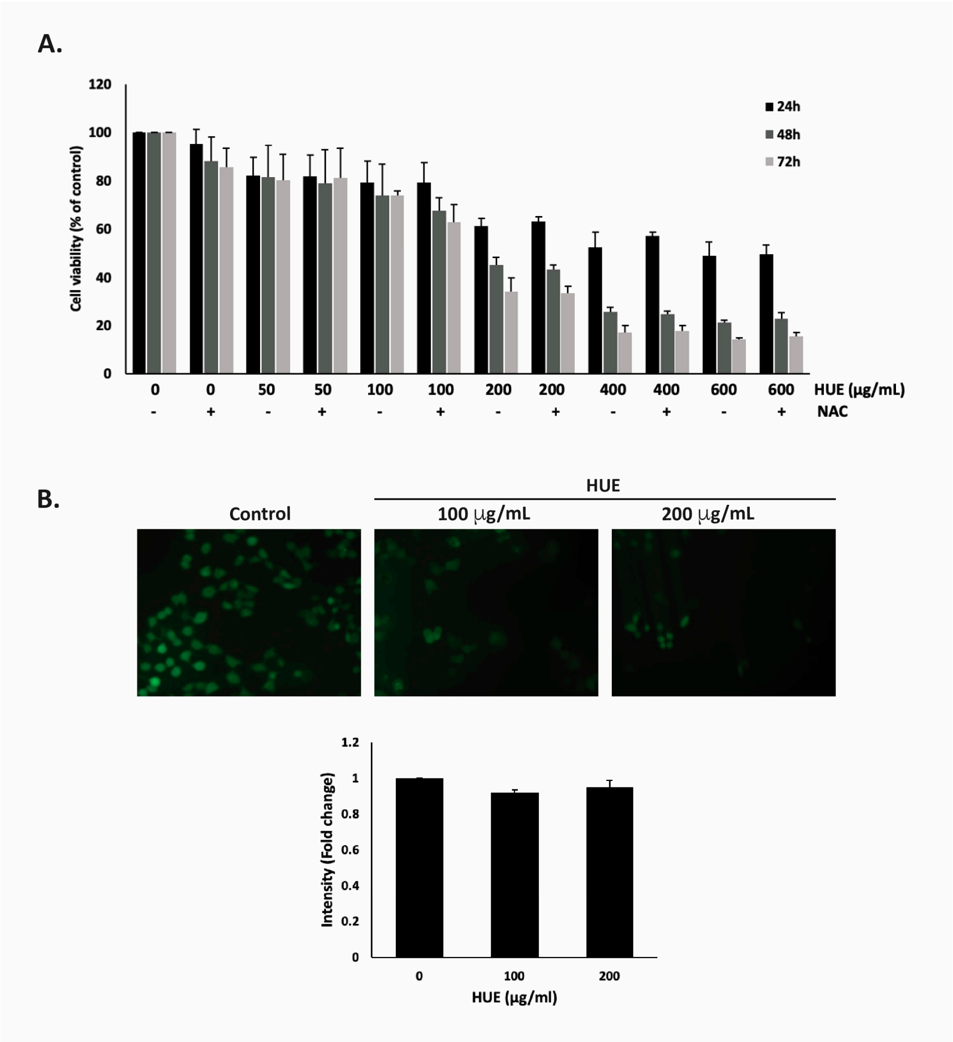

Figure S3. *Halodule uninervis* ethanolic extract does not induce oxidative stress in MDA-MB-231 breast cancer cells. (A) MDA-MB-231 cells were pre-treated with NAC (10 mM) for 30 minutes and then with the indicated concentrations of HUE. Cell viability was assessed using the MTT assay at 24, 48, and 72 h. HUE-treated cells without NAC pre-treatment were used for comparison. (B) Fluorescent images of 2’-7’-Dichlorodihydrofluorescein diacetate (DCFDA)-stained MDA-MB-231 cells. Cells were treated with or without the indicated concentrations of HUE for 24 h and stained with DCFDA to measure intracellular ROS production. Values represent the mean SEM of three independent experiments (n = 3) performed in triplicates and expressed as percentage of vehicle-treated control cells.
